# The Prognostic Value of Traditional Chinese Medicine Symptoms in Acute Ischemic Stroke: A Pilot Study

**DOI:** 10.1155/2020/1520851

**Published:** 2020-07-15

**Authors:** Jia Xu, Jian Pei, Qin-hui Fu, Yi-jun Zhan

**Affiliations:** Department of Acupuncture, LongHua Hospital, Shanghai University of Traditional Chinese Medicine, Shanghai, China

## Abstract

**Background:**

Stroke scales of traditional Chinese medicine (SSTCM) are promoted for use in the early prognosis. The current lines of evidence to support their performance evaluation are uneven. This pilot study aimed to investigate the correlation between traditional Chinese medicine (TCM) symptoms in the early stages of acute ischemic stroke and the prognosis of motor dysfunction through one-year of follow-up.

**Methods:**

Three hundred and fifteen patients were retrospected at Longhua Hospital from January 2016 to December 2017. All patients had received standard treatments combined with acupuncture therapy, including both electroacupuncture and scalp acupuncture for a median course of five months. The observed outcomes were the Fugl-Meyer assessment (FMA), the modified Barthel index (MBI), and the modified Rankin scale (mRS) at one-year follow-up after stroke onset by multiple linear regression analysis combined with ROC curves.

**Results:**

The favorable outcome rate was 74.3%, with the recurrence rate of 20.3% in the follow-up. In multiple linear regression, 10 TCM symptoms (MBI regression model) were related to the prognosis of MBI (DW 1.409, Ad. *R*^2^ 0.654) and 10 TCM symptoms (FMA regression model) were related to the FMA outcome (DW 1.446, Ad *R*^2^ 0.620). The two models were selected to have nine repeated symptoms (repeated model). In the ROC curves, the three models were compared with the NIHSS score, and the MBI regression model reflected the highest efficiency.

**Conclusions:**

The combination of 10 TCM symptoms, once onset occurred, including hemiplegia, restlessness, hemianesthesia, short breath, headache, constipation, night sweat, tinnitus, thirsty, and gurgling with sputum, may affect the recovery of motor dysfunction. Furthermore, the improvements of TCM symptoms dynamically after treatment would be observed in a large prospective cohort. This trial is registered with NCT01806233.

## 1. Background

Stroke is associated with the highest disability-adjusted life-years (DALYs) cost, as the first fatal disease in China, with over two million new cases annually [1], accounting for 40% of the global total [2]. With the change of environment and unhealthy lifestyle, the incidence among young and middle-aged adults (20–64 years old) displays an increasing trend [3]. The estimated onset age of stroke in China is about 10 years earlier than that in developed countries [1]. Typically, motor dysfunction is the most severe neurological deficit after stroke, with a disability rate of as high as 60% [4]. Given that China had the highest morbidity and mortality rates globally, more vigorous and effective actions are needed to reduce the burden of stroke.

Considering the high disability and recurrence rates of ischemic stroke, early prognosis plays an important role in making the treatment plans and evaluating clinical efficacy. With the progress of bio-psycho-social model of medicine, health-related quality of life (hr-QOL) and patient-reported outcomes (PROs) are regarded as the gold standards for evaluating the quality of life and subjective feelings of patients [5]. SSTCM, established under the guidance of TCM, and through the combined use of the four diagnostic methods, pay more attention to the unity of the body and spirit, pattern differentiation, and personal physique so as to adapt to Chinese conditions. However, the current evidence showed the performance evaluation of TCM scales is uneven [6]. Scales in general accepted standards have not been applied as early predictive indicators of stroke outcome in the prognostic scale [7]. Typically, some early prognostic symptoms are important for the development of precision medicine, quality of life, and prognosis of stroke, which need to be testified in the progress of medical practice.

With the rapid development of big data, real-world evidence is playing an increasing role in healthcare decisions. As a complementary role of clinical evidence, real-world data can come from various clinical practices directly, without deliberate selection of subjects and excessive manual intervention [8]. Consequently, real-world studies can reconstruct the real clinical scene maximumly in order to investigate clinical problems, especially in etiologic and prognoses research. This retrospective study, as one part of real-word studies, can provide evidence for evidence-based practice.

This pilot study aimed to investigate the correlation between TCM symptoms in the early stages of acute ischemic stroke and the prognosis of motor dysfunction. The clinical cases were retrospectively reviewed to assess the motor dysfunction in FMA through a one-year follow-up so as to provide evidence for the establishment of prognostic SSTCM.

## 2. Materials and Methods

### 2.1. Study Design

In this retrospective study, the inpatient electronic medical records database at Longhua Hospital Affiliated to Shanghai University of Traditional Chinese Medicine was accessed to analyze the secondary data.

### 2.2. Study Subjects

1650 cases with acute ischemic stroke were recruited between January 1^st^, 2016, and December 31^st^, 2017, from five inpatient departments in Longhua Hospital: department of TCM, neurology department, emergency ward, special ward, and geriatrics department. All patients received the standard treatments in accordance with the Chinese guidelines for the diagnosis and treatment of acute ischemic stroke [9].

### 2.3. Diagnostic Criteria

1. Ischemic stroke was diagnosed with reference to the criteria of cerebral arterial thrombosis from the Chinese Stroke Society [9]
2. TCM diagnostic criteria of apoplexy referred to the “Standards of Syndrome-Differentiated Diagnosis of Apoplexy” in Chinese medicine [10]

### 2.4. Inclusion Criteria

1. Adults patients should meet the diagnostic criteria of acute ischemic stroke and apoplexy mentioned previously
2. Ischemic stroke can be confirmed by a brain computed tomography (CT) or magnetic resonance imaging (MRI) scan
3. Patients who experienced an acute stroke within 24 h were included.
4. Stroke patients with limb motor dysfunction were recorded
5. The National Institutes of Health Stroke Scale (NIHSS) scores, the Fugl-Meyer Assessment (FMA), and TCM symptoms in acute state were completely recorded
6. Cases with correct contacts for follow-up were included

### 2.5. Exclusion Criteria

Cases with any of the following exclusion criteria were excluded: (1) patients with transient ischemic attack; (2) presence of another chronic severe disease, including myocardial infarction, heart failure, renal failure, malignant tumor, severe infection, Parkinson's disease, and epilepsy; (3) patients with conscious disturbance and the Glasgow Coma Scale (GCS) score of ≤8; (4) patients with severe dementia, to be specific, the Mini-Mental State Examination (MMSE) score of ≤17 in illiterate patients, that of ≤20 in patients receiving primary education, and that of ≤24 in those receiving junior or higher education; (5) patients and their families who were reluctant to be followed up.

### 2.6. Data Collection

Medical records, including identification (such as age, sex, and contact information), past medical history, examination, stroke onset time, admission time, NIHSS scores, FMA, and TCM symptoms and signs, were collected on the date of admission. All TCM symptoms were placed in a table in Excel, and those with frequencies greater than 5% were screened for further analysis. Each screened symptom had a score from 0 to 3 according to the severity. Typically, the electronic medical record system was designed by the Shanghai Kingstar Winning Software company (EMR No. 5, the architecture of Client/Server model). Participants were invited to the hospital for follow-up by telephone at one year after stroke onset.

### 2.7. Intervention

In this retrospective cohort study, all patients received standard treatments [11], including basic treatments for vital signs, antiplatelet medication, intravenous thrombolytics, and mechanical thrombectomy if eligible. Additionally, acupuncture therapy and rehabilitation were also carried out at the acute phase.

Acupuncture therapies, including electroacupuncture and scalp acupuncture, were also given at the acupoints of Hegu-LI4, Shenting-DU24, Fenglong-ST40, and Baihui-Du20 as well as the motor area of the scalp for 30 min once a day (5 days/week) in the hospital. Specifically, the electroacupuncture at the paretic side was divided into two protocols, including Jianyu-LI15, Quchi-LI11, Waiguan-TE5, Yanglingquan-GB34, and Zusanli-ST36 with dilatational wave (1.5∼2.5 Hz) protocol in the presence of flaccid paralysis; Tianjing-TE10, Naohui-TE13, Waiguan-TE5, Weizhong-BL40, and Chengshan-BL57 with dilatational wave (2 Hz) protocol for spastic paralysis. Sterile disposable needles (0.25  mm × 40  mm in size) were utilized in this study, which were inserted into the scalp acupoints as described in our prior protocol [12]. Briefly, the needles were twirled for 1 min (at least 200 turns per min) for once every 10 min.

### 2.8. Outcome Measurement

The primary outcome was the FMA scale at one-year follow-up after stroke onset. The FMA scale is a preferred, reliable, and stable tool for the assessment of motor functional performance of patients in restorative stroke trials [13], including flexor synergy, extensor synergy, movement combining synergy, movement out of synergy, coordination, and speed. The total store of 100 reflects a normal motor function.

The secondary outcomes were the MBI scale and the mRS scale at one-year follow-up after stroke onset. The MBI is a scale that measures ten basic activities of daily life, including dressing, feeding, continence of the bladder and bowel, going to the toilet, grooming, bathing, moving, walking at 45 m, and walking up and down stairs, which are related to self-care and mobility [14]. The normal scale is 100. In addition, the mRS scale is commonly used to measure the disability degree of patients with stroke or other neurological disability in daily activities, which has become the most extensively utilized clinical outcome measure in clinical studies [15]. The scale score ranges from 0–6 points. Favorable outcome was defined as a score from 0 to 2 on mRS at first year.

### 2.9. Statistical Analysis

Statistical analysis was carried out by the Statistical Product and Service Solutions (SPSS) statistical package program (version 25, SPSS Inc., Chicago, IL, USA). The quantitative data such as FMA, MBI, and NIHSS were expressed as means with standard deviation. The count data such as TCM symptoms were expressed as counts (percentage). Frequency comparisons were made with the methods of Chi-square test. Multiple regression analysis was used to determine the correlation between TCM symptoms and FMA. All variables were force-entered. Collinearity diagnostics were made to analyze multicollinearity. Durbin-Watson (DW) test was made to check the regression models. Moreover, a receiver operating characteristic (ROC) curve was drawn to verify the discriminatory power of the models.

## 3. Results

### 3.1. General Patient Characteristics

**Figure 1:**
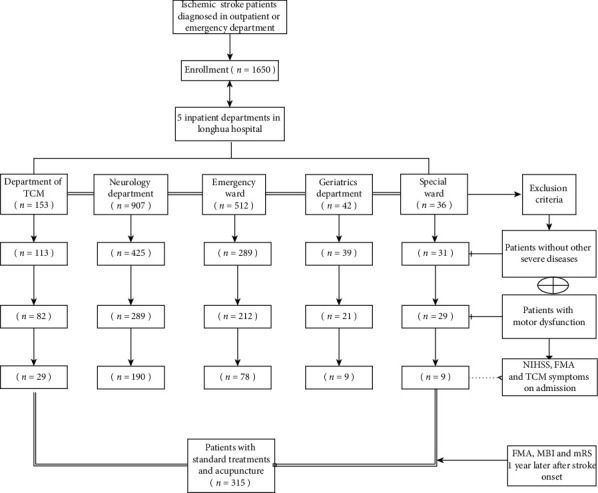
Flowchart of the study recruitment.

Eventually, 315 out of the 1650 patients at five inpatient departments of Longhua Hospital Affiliated to Shanghai university of TCM were enrolled in this retrospective cohort study in accordance with the inclusion and exclusion criteria from January 2016 to December 2017 (Figure 1).

In the overall sample, the mean age was 68.64 ± 10.72 years. 61.0% were males. 100 (31.7%) cases had a stroke history and 104 (33.0%) cases had a surgery history. In the past medical history, the first five diseases with high frequency were hypertension (76.8%), diabetes (42.5%), migraine (24.8%), coronary atherosclerotic heart disease (24.4%), and interstitial lung disease (16.5%), respectively. The mean scores of NIHSS, FMA, MBI, and mRS on admission were 7.51 ± 3.52, 30.65 ± 9.60, 30.66 ± 7.76, and 3.42 ± 0.63, respectively. The infarct locations mainly included basal ganglia (25.1%), internal capsule (18.2%), cerebellum (11.5%), frontal lobe (11.3%), and brain stem (10.8%).

In medication therapy, 25 (7.9%) patients received thrombolytic therapy. In antiplatelet medications, 33.8% took bayaspirin, 58.6% took clopidogrel, and 17.9% took ticagrelor. 10.3% especially took two kinds simultaneously. After discharge, the patients continued acupuncture treatment in the outpatient department. The median course of acupuncture treatment was five (five to six) months.

In the follow-up, the mean scores of FMA and MBI at the first year were 83.25 ± 14.73 and 81.79 ± 13.63. Favorable outcome (*n* = 234, 74.3%) was defined as a score from zero to two on mRS at the first year. No patients died. The overall recurrence rate was 20.3%.

### 3.2. The Correlation between TCM Symptoms and Motor Function at One-Year after Stroke

Twenty-five TCM symptoms on admission were screened totally. Using Chi-square test, 18 TCM symptoms were related with mRS at the first year, which showed their sensitivity to predict motor function of mRS (*P* < 0.05) (Table 1).

**Figure 2:**
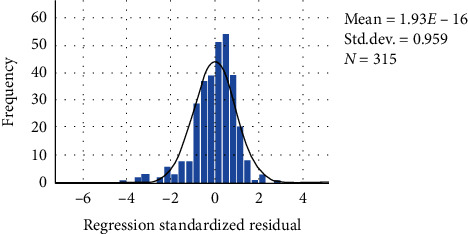
Regression standardized residual histogram (MBI).

**Figure 3:**
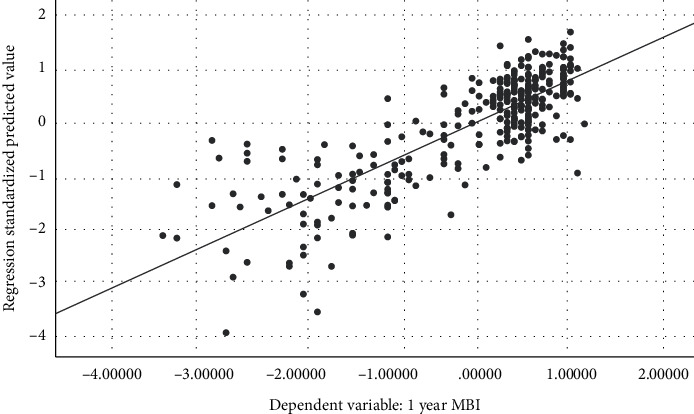
Regression plot (measured value vs. predicted value) (MBI). *R*^2^ = 0.682.

In multivariable linear regression analysis, 10 symptoms were proved to be statistically correlated with MBI at the first year (*P* < 0.05). The DW statistic was 1.409, which indicated nonautocorrelation. The R-squared value was 0.682, which indicated the goodness of fit. The adjusted R-squared was 0.654, which showed the regression model was reliable.  Figure 2 showed the analysis of residual.  Figure 3 showed a linear trend between the measured and predicted values of regression. The results of the standardized B value suggested that seven symptoms might be the primary symptoms to affect the prognosis of motor function, such as restlessness, hemiplegia, short breath, headache, and hemianesthesia (Table 2).

**Figure 4:**
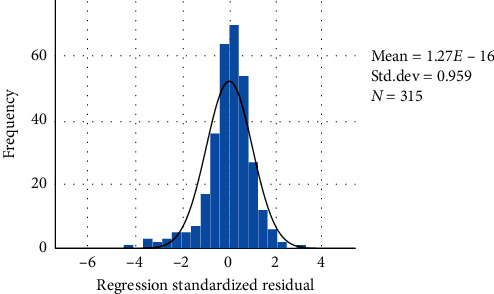
Regression standardized residual histogram (FMA).

**Figure 5:**
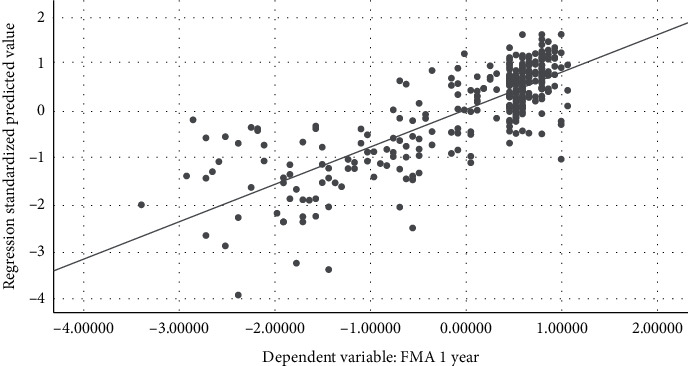
Regression plot (measured value vs. predicted value) (FMA). *R*^2^ = 0.650.

On the other hand, 10 symptoms were proved to be statistically correlated with FMA at the first year (*P* < 0.05) in multivariable linear regression analysis. The DW statistic was 1.446, which indicated nonautocorrelation. The *R*-squared value was 0.650, which indicated the quality of linear fit. The adjusted *R*-squared was 0.620, which showed the regression model was reliable.  Figure 4 showed the analysis of residual.  Figure 5 indicated a linear trend between the FMA and predicted values of regression. The results of the standardized B value suggested that seven symptoms might be the primary symptoms to affect the prognosis of motor function, such as restlessness, hemiplegia, short breath, headache, and constipation (Table 3).

**Figure 6:**
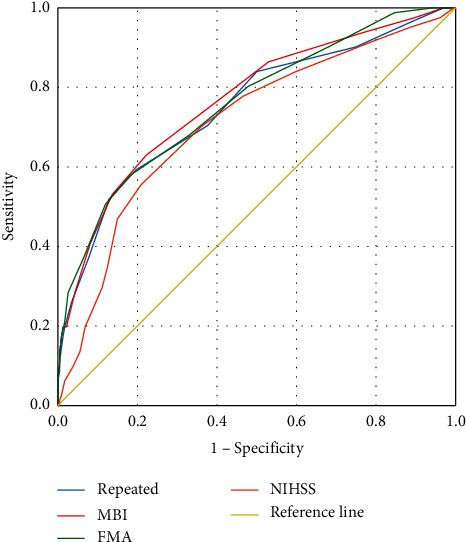
ROC curve on four scales.

After screening the above regression models (MBI and FMA), nine symptoms were of repeated occurrence, including hemiplegia, hemianesthesia, night sweats, short breath, headache, tinnitus, thirsty, constipation, and restlessness, which composed the third regression model. Moreover, the total scores of these three models were calculated, in contrast to the NIHSS scores at admission, respectively, so as to draw the ROC curve.  Figure 6 showed all the four curves were in the upper left corner closely. The results of area under the curve (AUC) showed that the sequence from high to low was MBI, FMA, repeated, and NIHSS, which indicated the higher accuracy of the three TCM scales. In sensitivity, the MBI model was the best (63%). In specificity, the FMA model was the best. Conclusively, the MBI model with 10 symptoms had the highest value in the prognosis of motor function (Table 4).

## 4. Discussion

In this paper, multivariable linear regression analysis was used to determine the correlation between 25 obtained TCM symptoms at admission and motor function by means of MBI and FMA at the first year. Moreover, ROC curves were drawn to verify the efficiency of the regression models.

The NIHSS scale is widely used to assess neurological impairment and evaluate the prognosis of motor function in acute stroke as well. The NIHSS scale consists of 11 items, including consciousness, eye movement, visual field, facial symmetry, body movement, mutual movement, sensation, and language function, with good validity and reliability [16]. However, some important factors closely related to prognosis are neglected, which might reduce the efficiency in evaluation [17]. The results indicated three TCM models with reasonable accuracy to have ROC curves in the upper left triangle above the reference line (Figure 6). Generally, the AUC can be thought of an indicator of overall “accuracy.” Considering an AUC of 0.767, the model of MBI with 10 symptoms had the highest value and best discrimination, although the specificity of the model of FMA is higher. Since the primary intended use of the prognostic test is either “ruling-in” or “ruling-out” a target condition, the balance between false-positive and false-negative rates is clinically important. In order to find the optimal trade-off between sensitivity and specificity, a value of ≥8 represents this point of balance, which indicates that the sensitivity in predicting motor dysfunction at the first year after stroke is 63% and the specificity is 77.8%. Meanwhile, while the critical value of NIHSS is nine, the sensitivity in predicting bad outcome is 55.6% and the specificity is 79.1%. Therefore, the TCM scale of MBI model is better in balance. In these 10 symptoms, hemiplegia is the primary symptom of stroke; hemianesthesia and headache are the specific symptoms of stroke; and the other seven symptoms, such as short breath, gurgling with sputum, constipation, and restlessness, are accompanied symptoms. For example, patients with restlessness (St. *B* = −0.286, 95% CI −0.371 to −0.202) on the first day were inclined to get poor recovery.

Generally, the ability to predict outcome in patients with acute stroke is of research and clinical value [18]. Previous stroke, age, and urinary or bowel incontinence on admission are adverse prognostic indicators of function [19]. Particularly, the severity of paresis, presence of hemianopia, reduced leg power, and size of brain lesion are all predictive of walking within 30 days after stroke [18]. Moreover, urinary incontinence, severity of hemiplegia, comorbidity, consciousness at admission, cognitive status, and depression are independent factors that are associated with the outcome in terms of activities of daily living (ADL) beyond six months [20]. The establishment of an adequate prognosis will increase the efficiency of stroke services and reduce costs. TCM symptom, as a kind of PROs, has been widely used in clinical practice for many years. The initial severity of specific symptoms and accompanied symptoms observed in the first day after stroke are important indicators of outcome at one year after stroke in this study.

Our previous studies [21, 22] found that wind syndrome and phlegm syndrome are the major single syndromes occurring within 30 days of onset. This study made further investigation into the prognostic value of definite symptoms occurring on the first day. For instance, gurgling with sputum, which is a reflection of phlegm syndrome, might be a factor affecting the recovery of motor dysfunction. A previous study [7] used the Pearson correlation test and logistic regression model to review 489 patients with acute ischemic stroke and finally screened eight items of the scale, indicating that early appearance of anxiety might predict a bad outcome at the ninetieth (90^th^) day (OR: 3.17). Our study observed the recovery of motor function at the first year, which was much longer. The symptoms collected were based on the theory of TCM differentiation. For the concept of holism, we took all the patient-reported symptoms as a whole, by the use of multivariable linear regression analysis accompanied with the ROC curve. Therefore, the results of the predictive model were reliable, which could provide a basis for the development of TCM scales.

Some limitations should be noted in this study. Considering that the study was retrospective, the quantity of the collected information depended on the quality of medical records. Inadequate information made it difficult to assess principal component analysis for classification. However, these TCM symptoms at the early stage of stroke might be valuable for the establishment of prognostic scales. Besides, we could not observe the improvements of TCM symptoms dynamically after treatment due to the limitation of the literature. The regression model here just reflected the first day of admission. We will focus on this part in further research.

## 5. Conclusion

It is observed in this study that ,through the one-year follow-up, once the ischemic stroke onset occurred, the combination of 10 TCM symptoms, including hemiplegia, restlessness, hemianesthesia, short breath, headache, constipation, night sweat, tinnitus, thirsty and gurgling with sputum, may affect the recovery of motor dysfunction. Of them, seven symptoms, including restlessness, hemiplegia, short breath, headache, hemianesthesia, constipation, and gurgling with sputum, might be the primary symptoms.

## Figures and Tables

**Table 1:** The correlation between TCM symptoms and mRS at the first year.

TCM symptoms	mRS (0∼2), *n* (%) *n* = 234	mRS (3∼5), *n* (%) *n* = 81	*P* Value
Hemiplegia	234 (100)	81 (100)	<0.001
Nausea and vomiting	163 (69.7)	71 (87.7)	<0.001
Hemianesthesia	43 (18.4)	52 (64.2)	<0.001
Lassitude	91 (38.9)	35 (43.2)	0.043
Slobbering	46 (19.7)	24 (29.6)	<0.001
Night sweat	175 (74.8)	27 (33.3)	<0.001
Short breath	37 (15.8)	36 (44.4)	<0.001
Headache	9 (3.8)	34 (42.0)	<0.001
Tinnitus	83 (35.5)	3 (3.7)	<0.001
Feverish palms and soles	97 (41.5)	13 (16.0)	<0.001
Bitter taste	161 (68.8)	61 (75.3)	<0.001
Gurgling with sputum	29 (12.4)	30 (37.0)	<0.001
Dry pharynx	57 (24.4)	12 (14.8)	0.011
Thirsty	87 (37.2)	11 (13.6)	<0.001
Frequent urination	107 (45.7)	29 (35.8)	0.007
Constipation	88 (37.6)	35 (43.2)	<0.001
Restlessness	73 (31.2)	69 (85.2)	<0.001
Poor appetite	139 (59.4)	59 (72.8)	0.031

Significant difference, *P* < 0.05. *P*values based on Chi-square test.

**Table 2:** The correlation between TCM symptoms and MBI at the first year.

TCM symptoms	n (%)	St. B value (95% CI)	*P* value
Hemiplegia	315 (100)	−0.178 (−0.255, −0.102)	<0.001
Restlessness	142 (45.1)	−0.286 (−0.371, −0.202)	<0.001
Hemianesthesia	95 (30.2)	−0.105 (−0.181, −0.029)	0.007
Short breath	73 (23.2)	−0.166 (−0.240, −0.093)	<0.001
Headache	43 (13.7)	−0.114 (−0.193, −0.035)	0.005
Gurgling with sputum	59 (18.7)	−0.071 (−0.142, −0.001)	0.048
Constipation	123 (39.0)	−0.087 (−0.161, −0.012)	0.023
Night sweat	202 (64.1)	0.109 (0.032, 0.186)	0.006
Tinnitus	86 (27.3)	0.111 (0.042, 0.180)	0.002
Thirsty	98 (31.1)	0.122 (0.051, 0.192)	0.001

St: standardized; CI: confidence interval; significant difference, *P* < 0.05.

**Table 3:** The correlation between TCM symptoms and FMA at the first year.

TCM symptoms	n (%)	St. B value (95% CI)	*P* value
Hemiplegia	315 (100)	−0.176 (−0.256, −0.095)	<0.001
Nausea and vomiting	234 (74.3)	−0.080 (−0.155, −0.005)	0.037
Hemianesthesia	95 (30.2)	−0.100 (−0.180, −0.021)	0.014
Short breath	73 (23.2)	−0.134 (−0.212, −0.057)	0.001
Headache	43 (13.7)	−0.115 (−0.198, −0.031)	0.007
Constipation	123 (39.0)	−0.097 (−0.175, −0.019)	0.015
Restlessness	142 (45.1)	−0.287 (−0.376, −0.198)	<0.001
Tinnitus	86 (27.3)	0.091 (0.018, 0.163)	0.015
Thirsty	98 (31.1)	0.099 (0.025, 0.173)	0.009
Night sweat	202 (64.1)	0.122 (0.041, 0.204)	0.003

St: standardized; CI: confidence interval; significant difference, *P* < 0.05.

**Table 4:** The results of ROC curve.

Model	AUC (95%CI)	Youden index	Critical value	Sensitivity (%)	Specificity (%)	*P* value
NIHSS	0.711 (0.644, 0.778)	0.347	9	55.6	79.1	<0.001
MBI	0.767 (0.705, 0.829)	0.408	8	63	77.8	<0.001
FMA	0.761 (0.698, 0.823)	0.396	9	58	81.6	<0.001
Repeated	0.749 (0.684, 0.815)	0.396	8	59.3	80.3	<0.001

AUC: area under the curve; CI: confidence interval; significant difference, *P* < 0.05.

## Data Availability

All data included in this study are available upon request through contacting the corresponding author.

## Abbreviations

ADL: Activities of daily living
AUC: Area under the curve
DALYs: Disability-adjusted life-years
DW: Durbin-watson
FMA: Fugl-Meyer assessment
GCS: Glasgow coma scale
hr-QOL: Health-related quality of life
MBI: Modified Barthel index
MMSE: Mini-mental state examination
mRS: Modified Rankin scale
NIHSS: National Institutes of Health Stroke Scale
PROs: Patient-reported outcomes
ROC: Receiver operating characteristic
SPSS: Statistical product and service solutions
SSTCM: Stroke scales of traditional Chinese medicine
TCM: Traditional Chinese medicine.
